# Comparison of Synthetic Computed Tomography Generation Methods, Incorporating Male and Female Anatomical Differences, for Magnetic Resonance Imaging-Only Definitive Pelvic Radiotherapy

**DOI:** 10.3389/fonc.2022.822687

**Published:** 2022-02-08

**Authors:** Laura M. O’Connor, Jae H. Choi, Jason A. Dowling, Helen Warren-Forward, Jarad Martin, Peter B. Greer

**Affiliations:** ^1^ Department of Radiation Oncology, Calvary Mater Hospital, Newcastle, NSW, Australia; ^2^ School of Health Sciences, University of Newcastle, Callaghan, NSW, Australia; ^3^ School of Mathematical and Physical Sciences, University of Newcastle, Callaghan, NSW, Australia; ^4^ Commonwealth Scientific and Industrial Research Organisation (CSIRO), Australian E-Health Research Centre, Herston, QLD, Australia; ^5^ School of Medicine and Public Health, University of Newcastle, Callaghan, NSW, Australia

**Keywords:** MRI radiotherapy planning, image-guided radiotherapy, synthetic CT, computer-assisted radiotherapy planning, rectum neoplasms, cervix neoplasms, endometrium neoplasms, anal canal neoplasms

## Abstract

**Purpose:**

There are several means of synthetic computed tomography (sCT) generation for magnetic resonance imaging (MRI)-only planning; however, much of the research omits large pelvic treatment regions and female anatomical specific methods. This research aimed to apply four of the most popular methods of sCT creation to facilitate MRI-only radiotherapy treatment planning for male and female anorectal and gynecological neoplasms. sCT methods were validated against conventional computed tomography (CT), with regard to Hounsfield unit (HU) estimation and plan dosimetry.

**Methods and Materials:**

Paired MRI and CT scans of 40 patients were used for sCT generation and validation. Bulk density assignment, tissue class density assignment, hybrid atlas, and deep learning sCT generation methods were applied to all 40 patients. Dosimetric accuracy was assessed by dose difference at reference point, dose volume histogram (DVH) parameters, and 3D gamma dose comparison. HU estimation was assessed by mean error and mean absolute error in HU value between each sCT and CT.

**Results:**

The median percentage dose difference between the CT and sCT was <1.0% for all sCT methods. The deep learning method resulted in the lowest median percentage dose difference to CT at −0.03% (IQR 0.13, −0.31) and bulk density assignment resulted in the greatest difference at −0.73% (IQR −0.10, −1.01). The mean 3D gamma dose agreement at 3%/2 mm among all sCT methods was 99.8%. The highest agreement at 1%/1 mm was 97.3% for the deep learning method and the lowest was 93.6% for the bulk density method. Deep learning and hybrid atlas techniques gave the lowest difference to CT in mean error and mean absolute error in HU estimation.

**Conclusions:**

All methods of sCT generation used in this study resulted in similarly high dosimetric agreement for MRI-only planning of male and female cancer pelvic regions. The choice of the sCT generation technique can be guided by department resources available and image guidance considerations, with minimal impact on dosimetric accuracy.

## 1 Introduction

Magnetic resonance imaging (MRI)-based radiotherapy treatment planning is an increasingly popular concept in radiation oncology. MRI affords greater soft tissue contrast for tumor and organ at risk (OAR) definition than computed tomography (CT), and MRI-based planning reduces the registration errors associated with supplementary image registration (1–3). To move away from the conventional use of CT in treatment planning, a synthetic CT (sCT) is created from the MRI, to facilitate MRI-based treatment planning. The sCT is an estimation of the electron densities of the tissues in the body, which allows for dose calculation in the radiation therapy treatment planning systems.

Larger pelvic treatment sites have received less attention in this area of work than prostate treatment sites, with previous larger pelvis sCT generation methods utilizing small groups of patient numbers and without consideration of the differences in male and female pelvic anatomy (4–7). This is also significant as the treatment volumes for colorectal and gynecological cancers traverse a more variable body contour and bony anatomy than prostate treatments. Rectum, anal canal, and gynecological treatments involve the treatment of larger and more variable body contour and bony anatomy than prostate treatments with differing prescription doses to the gross tumor volume, surrounding tissue deemed to be at high risk of tumor spread; the disease-positive nodes; and the surrounding local nodal volumes.

Several methods of sCT creation have been reported in the literature, which can be summarized into essentially four popular methods: bulk density assignment, tissue class density assignment, atlas-based, and computer learning (8). However, more commonly, the hybrid approaches of these methods are being utilized for greater accuracy (9, 10). The choice of the sCT generation technique can be guided by the need for extra resources, the ease of application, dosimetric accuracy, and image guidance considerations.

Bulk density assignment involves applying a single Hounsfield unit (HU) value to an entire volume, usually assuming water equivalency. This method may also differentiate between bone and tissue regions and apply a separate HU value to bone for greater accuracy. It is a relatively easy method to implement, usually manually performed, meaning that it requires minimal resources such as additional software or complex scanning protocols. The limitations of this method are that it does not take tissue inhomogeneity into account and it also may not generate realistic digitally reconstructed radiographs (DRRs) for treatment image guidance structures (1, 8). This method is currently utilized in brachytherapy treatment planning, in which image guidance is not a consideration and tissue inhomogeneity is not of great concern, given the relatively sharp dose fall off around the applicator (11). Bulk density assignment has also been investigated for prostate and brain treatments and has been utilized for sCT quality assurance measures (1, 12–14).

Tissue class density assignment is an extension of bulk density assignment, in which tissue inhomogeneity is addressed to some extent. This method involves using particular MRI sequences, such as a DIXON sequence, to classify body tissues into subtypes, i.e., muscle, fat, bone, and air. Each of these tissue subtypes is assigned an appropriate HU value (15). Tissue class density assignment has been a popular method of sCT creation, commonly combined with other atlas-based methods to improve bone region estimations, and has been utilized in commercial software (9, 10, 16). Previous applications of this method have focused mostly on the pelvic region (12, 17, 18).

An atlas-based approach involves comparing an MRI to a library atlas of co-registered CT and MRI pairs. The MRI scans in the atlas are non-rigidly registered to the acquired MRI scan, and the deformation matrix is applied to the corresponding CT pairs, to create the sCT. This approach can be performed using a single atlas pair, but has seen greater success for pelvis sites when multiple atlases are registered and combined with local weighted voting of atlas patch values, termed a hybrid multi-atlas approach (19–21). This method takes tissue inhomogeneity into account and can be used with image guidance. This method has successfully been translated to the clinic and has also been used for bone definition in commercial hybrid sCT generation products (9, 21–23).

Deep learning is an increasingly popular machine learning method for sCT generation, utilizing deep convolutional neural networks to convert an MRI into an sCT scan. Deep learning methods have been used successfully in the literature for pelvic and brain sCT creation, with the model outperforming other sCT generation methods (4, 24, 25). Models can be trained using CT/MRI pairs or can utilize unpaired MRI and CT data, thereby reducing the errors introduced in the registration process between the two images (4, 26, 27). Deep learning methods commonly utilize generative adversarial networks (GAN) composed of a generator and discriminator trained with paired CT/MRI data, such that the generator creates the sCT from the MRI, while the discriminator differentiates whether the image is real or fake, providing feedback to the generator. This continues until the discriminator can no longer determine that the image is synthetic (27). Unpaired generative methods utilize a cycle GAN model in which a single GAN network creates the sCT as described above, while a second GAN network converts the sCT image back to an MRI, and the difference between the images is fed back to the training loop (27). Similar to atlas-based methods, deep learning can be used for image guidance.

Each of these methods has its advantages and trade-offs in its accuracy, time, and ease of conversion. sCT methods have been predominantly developed to date for prostate and brain treatment sites, and previous comparisons of sCT generation methods have been performed for prostate treatments (28–30). More recently, a deep-learning method was developed using a multicenter anorectal cancer patient cohort (31).

This work provides a comparison of four major methods for the generation of synthetic CT: bulk density assignment, tissue class density assignment, hybrid multi-atlas, and deep learning sCT generation for a large dataset of male and female rectum, anal canal, cervix, and endometrium treatments. The sCT methods were compared with the conventional CT scan in terms of dosimetric impact on the treatment plan and mean error and mean absolute error in Hounsfield unit values.

## 2 Materials and Methods

### 2.1 Patient Data Collection

Ethics approval for the study was obtained through the local health district human research ethics committee (ref:17/06/21/3.02), and all patients gave informed consent to participate in the trial. MRI and CT datasets and treatment plans of 40 patients (20 male, 20 female) who received radiation treatment for histologically confirmed malignancy of either the rectum, anal canal, cervix, or endometrium were used for the study.

Patients were positioned with their legs flat using a CIVCO Vac-Lok bag (CIVCO Medical Instruments, IA, USA) for immobilization. CT scans were acquired on a SOMATOM Confidence CT scanner (Siemens Healthineers, Erlangen, Germany) at 120 kV with 2.0 mm slice thickness. Oral or intravenous contrast was administered at the request of the radiation oncologist. MRI scans were performed immediately following the planning CT scan, on a MAGNETOM Skyra 3T MRI scanner (Siemens Healthineers, Erlangen, Germany), equipped with a Qfix flat couch (Qfix, PA, USA) and DORADOnova MR 3T external laser bridge (LAP, Luneburg, Germany). A 32-channel spine coil was utilized under the flat couch top and two 18-channel body coils were used over the pelvic region. To avoid compression of the external body contour, body coils were positioned in a Qfix INSIGHT MR Body coil holder. A stitched T1 VIBE Dixon MRI sequence (**Table 1**) was acquired to facilitate sCT generation. The Dixon imaging technique provides an in-phase, out-of-phase, fat-weighted, and water-weighted image from a single acquisition. The MRI and CT scan range included the entire lumbar spine to mid femur, and patients were scanned with a full bladder and empty bowels.

**Table 1:** MRI acquisition parameters.

Parameter	T1 VIBE Dixon
Scan type	VIBE Dixon
TE (ms)	1.23/2.46
TR (ms)	4.19
Flip angle (°)	9
FOV (mm)	256 * 499
Slice thickness (mm)	1.6
Base resolution	160
Acquisition plane	Coronal
Phase direction	R>L
Bandwidth (Hz/px)	1,200
Fat-water shift (px)	0.3
Distortion correction	3D
Acquisition stages	2
Overlap (mm)	48
Composing	Inline

Treatment planning was performed on the CT scan using the Eclipse TPS (version 15.6; Varian Medical Systems, Palo Alto, USA). Three patients in the male cohort were planned as intensity-modulated radiation therapy (IMRT), while all the other patients were planned as 6-MV, 2–3 arc volumetric-modulated arc therapy (VMAT). The three patients planned as IMRT were re-planned as VMAT retrospectively, to standardize the planning technique analyzed for the study.

### 2.2 sCT Creation

#### 2.2.1 Bulk Density Assignment

The bulk density assignment included two tissue classes—bone and soft tissue. The bone regions were outlined manually on the T1 in-phase Dixon MRI sequence, while the whole body region was defined by image thresholding. Choi et al. had previously derived the optimal bulk density values for bone and tissue to patients treated for prostate cancer, which equated to a relative electron density of 1.20 and mass density of 1.25 g/cm**^3^** for bone regions and a relative electron density of 0.97 and mass density of 0.99 g/cm**^3^** for tissue (12).

#### 2.2.2 Tissue Class Density Assignment

For the tissue class density assignment method, the tissue was separated into three tissue classes: fat, muscle/visceral, and bone. The entire body region was defined using image thresholding and the bone regions were outlined manually on the T1 in-phase Dixon MRI sequence. The fat tissue was segmented from the fat-weighted Dixon MRI image using image thresholding. The muscle and visceral tissue was defined by a Boolean subtraction of the fat and bone regions from the body contour. The optimal electron densities of each tissue class in this study were also determined by Choi et al. for prostate treatments, which equated to a relative electron density of 1.16 and mass density of 1.20 g/cm**^3^** for bone regions, a relative electron density of 1.02 and mass density of 1.03 g/cm**^3^** for muscle, and a relative electron density of 0.91 and mass density of 0.92 g/cm**^3^** for fat (12).

#### 2.2.3 Hybrid Multi-Atlas Based

Participants were separated into male (*n* = 20) and female (*n* = 20) cohorts for the creation of gender-specific atlases. A bias field correction was applied to the MRI image to homogenize the image intensity across the field, and the hybrid atlas-based sCT generation method of Dowling et al. was utilized, with a leave-one-out cross-validation approach applied for both groups (i.e., 19 CT/MRI pairs were used to generate an sCT for each target patient MRI) (21). This approach was modified from the original method to account for the larger field of view, by utilizing a custom structure-guided rigid (6 degrees of freedom) and non-rigid registration (using binary labels based on the bone and bladder contours) between each CT and MRI pair in the atlas set. Each MRI in the atlas set was registered to the acquired MRI initially by using the body mask, and then deformably registered to the target MRI. A local weighted voting was then applied with a 3D radius and a gain parameter to increase the sensitivity of patch value similarities. These weightings were applied to the corresponding patches in each of the co-registered CT scans in the atlas to create the sCT.

#### 2.2.4 Deep Learning

The deep learning model was created using a conditional generative adversarial network (cGAN), consisting of a single generator and a discriminator, trained using the paired MRI-CT data (32). The network is set with a condition, meaning that both the generator and the discriminator of the network are conditioned on the CT (target) image for a direct MRI to sCT conversion (32). The CT and MRI scans were preprocessed using a binary mask to remove the background and the scans were resampled to a matrix of 320 × 320 voxels. A bias field correction was applied to the MRI image to homogenize the image intensity across the field, and then standardization of the image intensity peaks was applied to standardize tissue weightings across the whole cohort. Image registration between the CT and MRI was performed using structure-guided (bone) non-rigid registration. The patient cohort was separated into four groups of 10 and then used for training and testing four individual cGAN models using four-fold cross-validation, that is, each model was trained with the CT/MRI pairs of 30 patients and generated sCT scans of 10 patients for testing.

**Figure 1:**
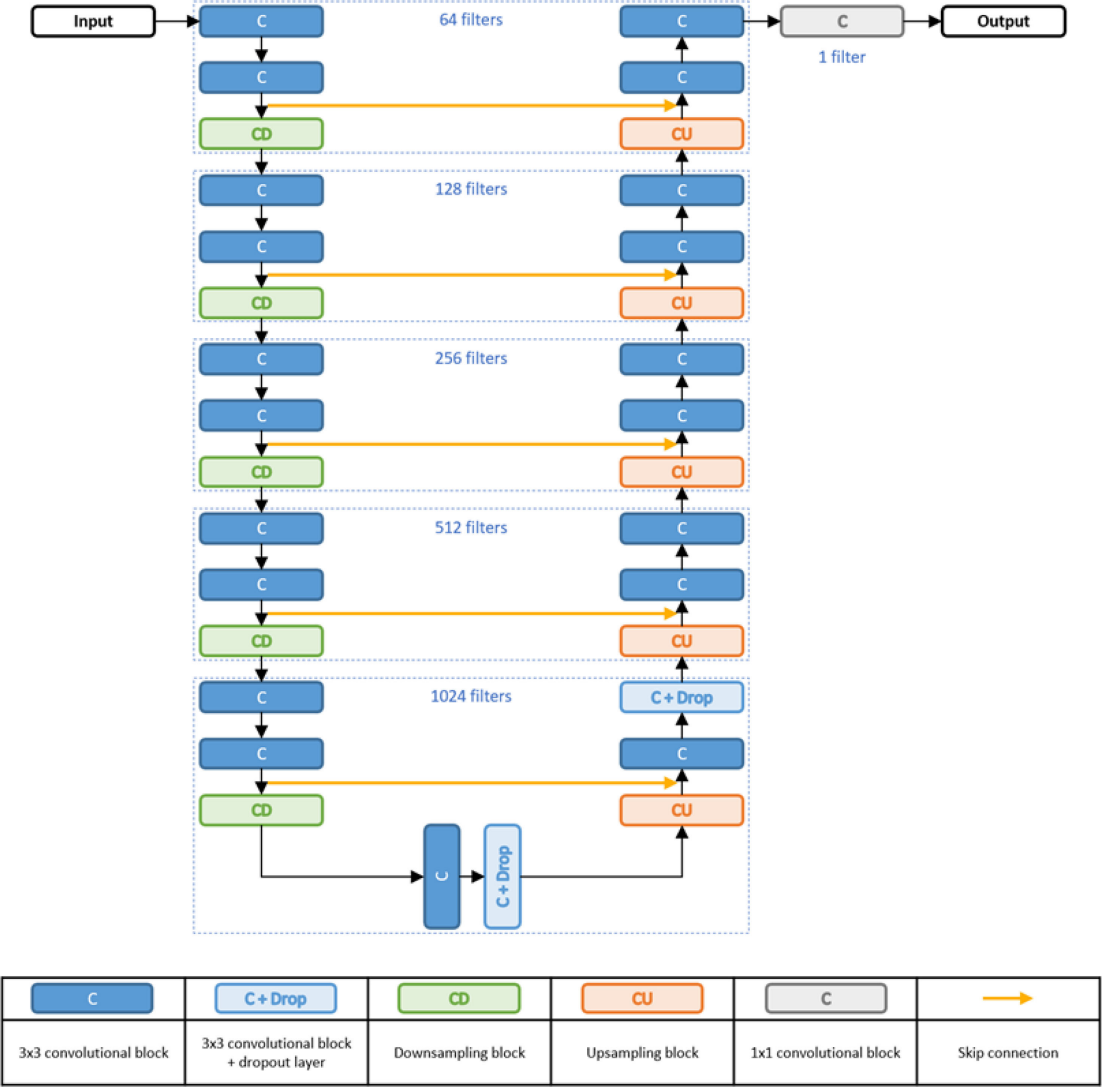
Generator architecture. Note that the number of filters (i.e., 64 filters) indicates the number of filters in each convolutional layer.

The generator was a modified U-net with a similar architecture to the model proposed by Han et al. and Largent et al. (**Figure 1**) (26, 30). The network was an encoding–decoding network that extracted features from the input MRI and reconstructed an sCT using these features. At the encoding part, features were extracted through the convolutional blocks with a filter size of 3 × 3 and stride 1. The features were then down-sampled through the down-sampling convolutional blocks with a filter size of 3 × 3 and stride 2, as suggested by Largent et al. (30). The features from the encoding part were then used in the decoding part to construct the sCT. At the decoding part, the feature maps were up-sampled *via* a 3 × 3 transpose convolution with stride 2, also suggested by Largent et al. (30). The last convolution block of the decoder part consisted of a 1 × 1 convolution with stride 1, followed by a hyperbolic tangent activation to output the sCT. In all except the last block, each convolutional block in the generator used batch normalization and a rectified linear unit (ReLU) activation function. Skip connections were added between the encoder and the decoder for concatenating the channels of the feature map. Furthermore, two dropout layers with a drop rate of 0.5 were applied, one after the bottleneck and the other after the CU1024 block to prevent overfitting.

The discriminator was a small CNN-based image classifier, and its architecture was similar to the model proposed by Largent et al. (30). It can be defined as:

Discriminator:CD8-CD16-CD32-CD64-CD128-C128-F where CD was the down-sampling block and F was the fully connected layer with sigmoid activation. The numbers on each block indicate the number of filters. All the CD blocks of the discriminator used the leaky ReLU, with the slope coefficient 0.2, as the activation function for allowing a small gradient for the input that is less than zero (33). The last convolutional block (C128) operated a 1 × 1 convolution with stride = 1. The output was then fully connected for 1D output. The discriminator was a binary classification model using binary cross entropy so that it predicts an output in the range of 0 (fake) and 1 (real).

The weights of both models were initialized with a normal distribution with mean = 0 and standard deviation = 1e-4. For updating the generator, a loss was calculated by adding the adversarial loss, calculated *via* the discriminator, and the L1 (mean absolute error) loss between the generated output (sCT) and the target image (CT). Weights on both the adversarial and the L1 losses were set to 1. Adam optimization algorithm was used for stochastic gradient descent optimization and the learning rate was set to 1e-4. Batch size and training epochs were set to 10 and 20, respectively.

### 2.3 sCT Validation

The CT scan, T1 VIBE Dixon MRI, and sCT datasets were imported into the treatment planning system. For each subject, the atlas-based and deep learning sCT datasets were co-registered to the MRI and the MRI registered to the CT using rigid registration, with a registration boundary of the top of the second lumbar vertebrae to the greater trochanter. The bulk density and tissue class segmentation methods were performed on the MRI scan. To reduce disparity introduced by bowel gas, the bowel gas on the datasets was overridden to average surrounding tissue HU value for 10 patients. Two patients with a large discrepancy in body contour of >4 cm in the lateral posterior region, between the CT and MRI, due to tensing of the gluteal muscles in CT, had this region removed from the sCT calculation volume, so as to not affect the results.

The treatment plan, structure set, and International Commission of Radiation Units and Measurements (ICRU) reference point were copied from the original CT to each sCT, and the plan was recalculated with identical monitor unit values. Dosimetry was compared using the dose difference at the plan ICRU point, relevant dose volume histogram (DVH) dose parameters for planning target volume (PTV) and organ at risk (OAR) structures per standard guidelines for each treatment site, and a 3D dose gamma comparison (34, 35). The plan on the CT scan was used as the gold standard. As several DVH parameters were evaluated for PTV and OAR structures, the average dose difference is a combined average of each of these parameters for all structures. The percentage dose difference was calculated by the formula (DsCT − DCT)/DCT * 100%. Due to the non-parametric nature of the data, statistical significance was determined using a Mann–Whitney ***U***-test with a significance level of 0.05. Three-dimensional gamma analysis was performed using an in-house MATLAB code (MATLAB; MathWorks, Massachusetts, USA), using dose difference (%) and distance to agreement (mm) criteria of 3%/2 mm, 2%/2 mm, and 1%/1 mm. An erosion of 15 mm of the body perimeter was applied to exclude failures which occurred at the skin edge due to small unavoidable differences in body contour between datasets, and a 10% low-dose threshold was applied.

Hounsfield unit estimation accuracy was assessed using mean error (ME) and mean absolute error (MAE) in HU value for the entire body and bone region. To account for regions of image degradation in some sCT datasets, the superior and inferior 30 mm of the datasets was not included in these calculations.

## 3 Results

Detailed patient demographics are outlined in **Table 2**. Of the 40 patient datasets used in the trial, 4 patients in the male cohort and 11 patients in the female cohort received iodine-based oral contrast, while 1 patient in the female cohort received iodine-based IV contrast for the planning CT scan.

**Table 2:** Patient demographics.

	Cohort size	Age range	BMI range (kg/m^2^)	Relevant surgical history	Primary treatment site	Staging range
**Male cohort**	20	49–88 (mean = 65)	20.5–33.6 (mean = 25.5)	Hernia repairs (*n* = 3) Rectal resections (*n* = 2) Appendectomy (*n* = 1)	Rectum (*n* = 20)	T1N0–T4N1
**Female cohort**	20	41–85 (mean = 61)	18.0–36.9 (mean = 26.2)	Hysterectomy (*n* = 6) Common iliac stent (*n* = 1) Caesarean (*n* = 1) Hernia repair (*n* = 2) Appendectomy (*n* = 3)	Rectum (*n* = 4)	T3N0–T3N2
					Anal canal (*n* = 4)	T1N0–T3N1
					Cervix (*n* = 8)	IIA–IIB
					Endometrium (*n* = 4)	IIIA–IIIC

**Figure 2:**
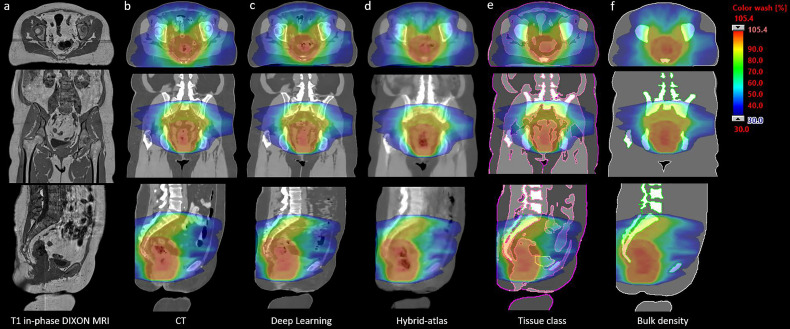
CT and sCT scans (with corresponding MRI) with treatment plan calculated and dose color wash displayed. Column **(A)** T1 in-phase-stitched T1 VIBE Dixon; column **(B)** original CT scan column; column **(C)** deep learning-generated sCT; column **(D)** hybrid atlas-generated sCT; column **(E)** tissue class density assignment sCT (three tissue classes); and column **(F)** bulk density overrides sCT.

All sCT generation methods were successfully applied to the MRI scan of each patient. An example of the conventional CT scan, T1-weighted MRI, and each sCT generation method for a single patient is shown in **Figure 2**. The closest agreement in ME and MAE in HU estimation was for the deep learning and hybrid atlas techniques for the whole body, bone, and soft tissue estimations (**Table 3**). The dosimetric results correlated well with the ME and MAE results, with the greatest difference in ME and MAE resulting in the greatest dosimetric error for the bulk density method.

**Table 3:** ICRU median percentage dose difference and median DVH dose difference by the sCT method.

	ICRU %DD		DVH %DD		Mean absolute error (HU)			Mean error (HU)		
	Median (IQR)	*p*-value	Median (IQR)	*p*-value	Whole body	Bone	Soft tissue	Whole body	Bone	Soft tissue
**Deep learning**	−0.03 (0.13, −0.31)	1.00	0.18 (0.40, −0.05)	0.93	34.7 ± 5.1	109.4 ± 12.3	25.2 ± 3.4	−2.5 ± 5.8	−46.0 ± 19.6	−0.7 ± 6.3
**Hybrid atlas**	−0.30 (−0.02, −0.57)	0.82	−0.27 (0.12, −0.77)	0.76	57.4 ± 8.0	186.9 ± 17.9	47.3 ± 7.8	−2.0 ± 9.0	−78.0 ± 35.3	4.1 ± 8.5
**Tissue class**	−0.48 (−0.28, −0.85)	0.68	−0.48 (0.11, −0.66)	0.71	58.8 ± 10.4	228.2 ± 11.2	44.6 ± 8.5	−9.8 ± 7.3	−25.8 ± 39.7	−8.6 ± 7.5
**Bulk density**	−0.73 (−0.10, −1.01)	0.64	−0.33 (0.19, −0.67)	0.70	89.1 ± 7.7	244.1 ± 10.0	76.1 ± 6.7	8.0 ± 13.7	5.7 ± 39.3	7.8 ± 14.7

Mean absolute error and mean error in whole body and bone HU ± 1 SD by the sCT method.

**Figure 3:**
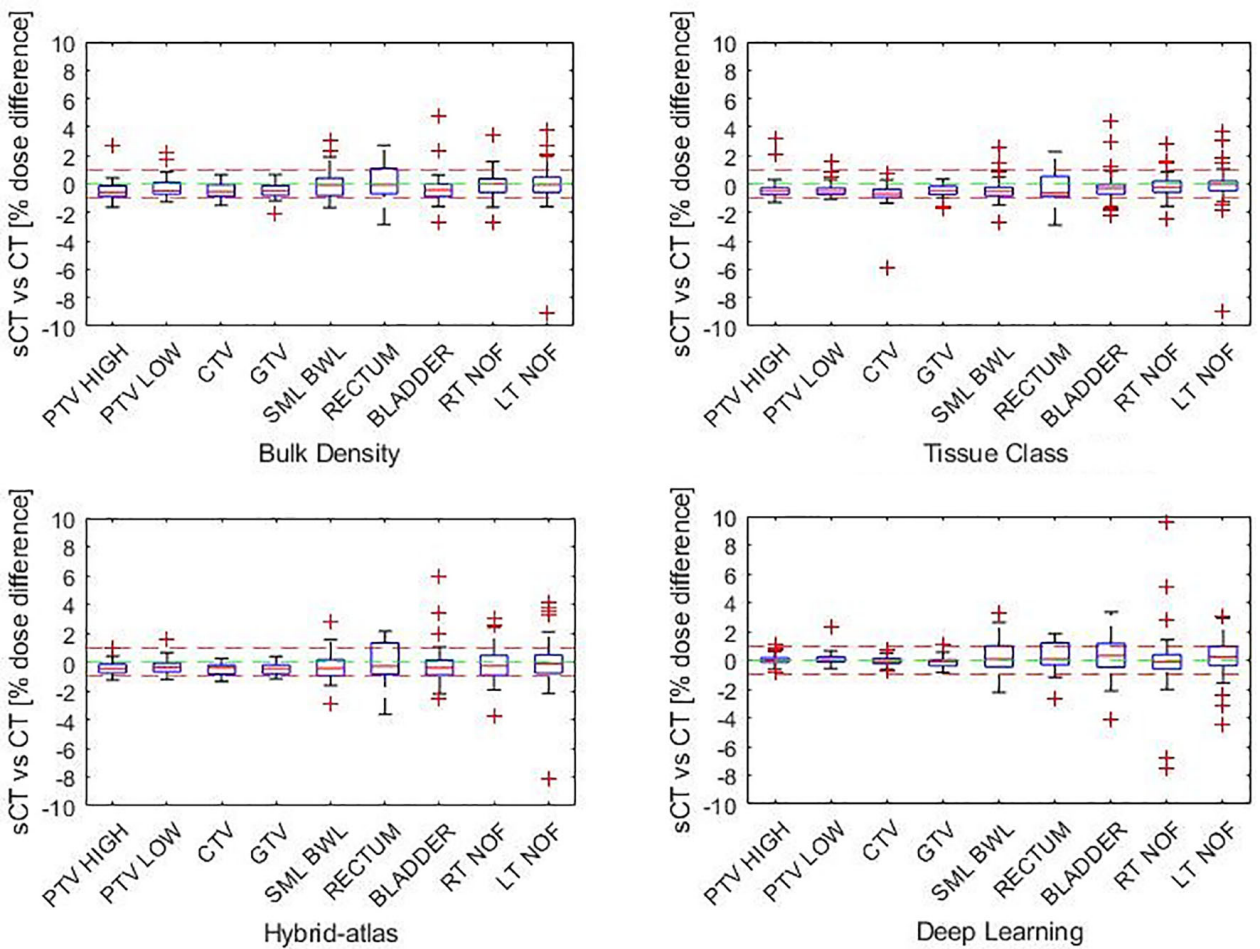
Percentage DVH dose difference by structure (each structure parameter combined) for each synthetic CT method.

There was no statistically significant dose difference to CT at the ICRU reference point for any of the sCT methods (**Table 3**). The median DVH dose difference for all structures and parameters combined was less than 0.5% for all sCT methods, with the greatest agreement for the deep learning method and the least agreement in the bulk density method (**Table 3** and **Figure 3**). There was a statistically significant difference (*p* = 0.002) for the bulk density sCT, with a median percentage dose difference at the ICRU reference point between the male and female cohort of −0.89% [interquartile range (IQR) of −0.72, −1.15) and −0.09% (IQR of 0.09, −0.83), respectively. There was no statistically significant difference in the median percentage dose difference at the ICRU reference point between the male and female cohort for the other sCT methods.

The 3D gamma results with criteria of 3%/2 mm for all sCT methods were within the American Association of Physics in Medicine (AAPM) TG218 report guidelines of >95% (**Table 4**) (36). The closest agreement at 1%/1 mm was seen for the deep learning sCT, while the greatest difference between the male and female cohort was in the hybrid atlas-based and bulk density methods.

**Table 4:** 3D gamma dose comparison for each sCT technique (mean ± 1 SD).

		3%/2 mm		2%/2 mm		1%/1 mm	
		Pass rate (%)	Average gamma	Pass rate (%)	Average gamma	Pass rate (%)	Average gamma
Deep learning	All	99.8 ± 0.3	0.09 ± 0.02	99.7 ± 0.4	0.14 ± 0.03	97.3 ± 2.0	0.28 ± 0.07
	Female	99.8 ± 0.3	0.10 ± 0.02	99.6 ± 0.4	0.14 ± 0.03	97.4 ± 1.2	0.28 ± 0.06
	Male	99.9 ± 0.3	0.09 ± 0.03	99.7 ± 0.4	0.14 ± 0.03	97.1 ± 2.5	0.27 ± 0.08
Hybrid atlas	All	99.8 ± 0.3	0.12 ± 0.04	99.7 ± 0.3	0.17 ± 0.05	94.8 ± 4.5	0.35 ± 0.11
	Female	99.8 ± 0.3	0.13 ± 0.04	99.7 ± 0.4	0.19 ± 0.06	93.4 ± 5.2	0.38 ± 0.12
	Male	99.8 ± 0.2	0.10 ± 0.03	99.7 ± 0.3	0.15 ± 0.04	96.3 ± 3.2	0.31 ± 0.09
Tissue class	All	99.8 ± 0.3	0.12 ± 0.03	99.7 ± 0.4	0.18 ± 0.04	95.3 ± 3.3	0.35 ± 0.08
	Female	99.8 ± 0.3	0.12 ± 0.02	99.7 ± 0.4	0.18 ± 0.03	95.2 ± 3.1	0.36 ± 0.07
	Male	99.9 ± 0.2	0.12 ± 0.03	99.7 ± 0.4	0.17 ± 0.04	95.4 ± 3.2	0.34 ± 0.09
Bulk density	All	99.8 ± 0.3	0.14 ± 0.03	99.7 ± 0.4	0.19 ± 0.05	93.6 ± 3.8	0.38 ± 0.09
	Female	99.8 ± 0.4	0.12 ± 0.03	99.6 ± 0.4	0.17 ± 0.05	95.1 ± 3.3	0.34 ± 0.09
	Male	99.9 ± 0.2	0.13 ± 0.03	99.7 ± 0.4	0.21 ± 0.05	92.1 ± 3.6	0.42 ± 0.09

## 4 Discussion

The results presented in this article compare favorably to previous studies of sCT generation for MRI-only planning. The methods chosen for sCT generation in this article were based on previous literature, which returned similarly high dosimetric agreement in separate studies on MRI-only planning for pelvic treatments. The bulk density assignment was based on the bone–tissue maps of Choi et al., which was applied to 54 prostate treatment plans (12). Choi et al. presented a dose agreement at ICRU point of −0.15% ± 0.90% (IQR = 0.31, −0.65) and a mean 3D gamma agreement of 90.7% ± 0.2% with a criteria of 1%/1 mm (12). The bone and body contours in this study were assigned the same densities to the study of Choi et al., to determine if those values are also applicable to male and female patients with anal canal, rectal, endometrial, or cervical cancer (12). Compared with the study of Choi et al., the dose agreement at ICRU point in this study was lower at −0.73% ± 0.59%, while the mean 3D gamma agreement at 1%/1 mm was higher at 93.6% ± 3.8%. The difference in the results may be due to the study of Choi et al. being optimized for prostate treatments, while this study applied the method to male and female cohorts with treatment regions in the pelvis and lower abdominal region (12). The bulk density values of the tissue could have been affected by a difference in fat to water ratio in this region and body mass index between the different cohorts of patients (37).

The fact that the density values were not re-optimized for the cohort of this study would also explain the difference in the results for the tissue class density assignment results. The tissue class density assignment method was based on the bone–muscle–fat (BMF) maps of Choi et al. which were once again applied to 54 prostate treatment plans (12). Compared with the cohort in this study, the dose agreement at the ICRU point was higher for Choi et al. −0.16% ± 0.65% (IQR 0.22, −0.60), compared with −0.48% ± 0.44% (IQR −0.28, −0.85), while the mean 3D gamma agreement at 1%/1 mm was lower at 93.8% ± 8.6% than in this study at 95.3% ± 3.3%.

The atlas-based method was based on the hybrid atlas sCT generation method of Dowling et al., which had previously shown to outperform the atlas-based method of the group (20, 21). Dowling et al. applied the hybrid atlas approach to 39 prostate cancer treatment plans. The hybrid atlas approach was modified in our study, for a larger anatomical region by incorporating structure-guided rigid and non-rigid registration (bone and bladder) in the atlas set. The results of the study of Dowling et al. correlate strongly with the results of this study, with similarly low percentage difference of the dose calculated at the ICRU reference point of −0.3% ± 0.8% and −0.3% ± 0.5%, respectively, and a mean 3D gamma agreement at 1%/1 mm of >95% and 94.8%, respectively (21).

The deep learning approach to sCT generation in this study is similar to that of Largent et al. and Maspero et al. (4, 30). The cGAN approach was favored for our study over a cycle GAN due to the high GPU memory requirements and long training times of the cycle GAN method. Maspero et al. applied the cGAN approach to sCT generation for pelvic radiotherapy, performing dose analysis on 30 patients (10 prostate, 10 rectum, and 10 cervix) (4). Maspero et al. reported a similar dose difference of 0.1%–0.3% to this study of −0.03% ± 0.42% and a gamma agreement at 2%/2 mm of 94.8% compared with this study of 99.7% ± 0.4%.

All sCT generation methods assessed in this study returned similarly high dosimetry agreement when compared with CT and were all within clinically acceptable ranges. However, image guidance and the amount of resources required are other drivers in the choice of an sCT generation method. Accurate bony anatomy is required for image guidance on treatment. Although bulk density and tissue class density assignment methods do not necessarily require additional software to perform, the manual contouring of the bone regions on MRI can be time-consuming. As such, these methods have successfully been combined with an atlas-based approach for the bone mask and are the basis of commercial sCT generation products (9, 10). Atlas-based and deep learning methods both provide reliable bone definition and are increasingly favored for MRI-only planning due to image guidance considerations. Deep learning methods have the advantage over atlas-based methods in the time it takes to generate an sCT. The deep learning method presented in this study took 3.5 s for a single sCT generation, while the atlas-based method took approximately 50 min. Therefore, for the methods presented in this study, there are advantages and drawbacks of each method, and centers are able to use this knowledge to identify the most suitable method for MRI-only planning.

This study has shown that a bulk density assignment, tissue class segmentation, hybrid atlas, and deep learning methods of sCT generation can be utilized for MRI-only planning of male and female cancers of the rectum, anal canal, cervix, and endometrium. The implications of this study indicate that selection of an sCT generation technique can be driven by department resources, with minimal impact to plan dosimetry, therefore greatly expanding the accessibility of MRI-only planning in radiation therapy.

## Data Availability Statement

The datasets presented in this article are not readily available because ethics approval for this study does not allow for sharing of individual patient scans. Other study data, which do not include patient datasets, are available from the corresponding author on reasonable request. Requests to access the datasets should be directed to Laura.OConnor@calvarymater.org.au

## Ethics Statement

The studies involving human participants were reviewed and approved by Hunter New England Human Research Ethics Committee. The patients/participants provided their written informed consent to participate in this study. Written informed consent was obtained from the individual(s) for the publication of any potentially identifiable images or data included in this article.

## Author Contributions

LO’C was involved in the study design, patient recruitment, data collection, data analysis and wrote the manuscript. JC developed the deep learning-based synthetic CT creation method used in this study. The tissue class density assignment and bulk density synthetic CT methods were based on previous work by JC. JD developed the atlas-based synthetic CT creation method used in this study. JD, JC, PG, HW-F, and JM contributed to the study design data analysis and revised the manuscript critically for important intellectual content. JM contributed to the formal analysis. All authors read and approved the final manuscript.

## Funding

LMO, PG, JD and JM received research grant funding through the Calvary Mater Newcastle Hospital (Margaret Mitchell Research grant 16-12) for the development of atlas-based algorithms. JHC and PG received research grant funding through the Australian National Health and Medical Research Fund (The Australian MRI-Linac Program) for the development of deep learning algorithms.

## Conflict of Interest

The authors declare that the research was conducted in the absence of any commercial or financial relationships that could be construed as a potential conflict of interest.

## Publisher’s Note

All claims expressed in this article are solely those of the authors and do not necessarily represent those of their affiliated organizations, or those of the publisher, the editors and the reviewers. Any product that may be evaluated in this article, or claim that may be made by its manufacturer, is not guaranteed or endorsed by the publisher.
